# Hereditary Vitamin-D Dependent Rickets Type II: A Case Report

**DOI:** 10.31729/jnma.6411

**Published:** 2021-06-30

**Authors:** Neela Sunuwar, Swotantra Gautam, Anu Radha Twayana, Saroj Adhikari Yadav, Firoz Anjum, Kriti Kandel

**Affiliations:** 1 B.P. Koirala Institute of Health Sciences, Dharan, Nepal; 2 Kathmandu University School of Medical Sciences, Dhulikhel, Nepal; 3 Department of Paediatrics, Patan Academy of Health Sciences, Lalitpur, Nepal

**Keywords:** *cinacalcet*, *rickets*, *vitamin D*

## Abstract

Hereditary vitamin D dependent rickets type II is a rare genetic disorder in children characterized by early onset of rickets and deranged biochemical parameters. Low serum calcium level, high alkaline phosphatase, high parathyroid hormone, and high values of 1,25-dihydroxy vitamin D are characteristic biochemical findings. We are reporting a rare case of Vitamin D Dependent Rickets and subsequent improvement after addition of cinacalcet. This is a case report of a 2.5-year-child with Hereditary Vitamin D Dependent Rickets type II receiving cinacalcet as adjunct to oral calcium and calcitriol. Oral cinacalcet (0.25mg/kg/day) was added to the regimen as an adjunct after treatment failure with high dose of oral calcium and calcitriol. A significant improvement in radiological findings and normal homeostasis of calcium, phosphate and parathyroid hormone was achieved after initiation of cinacalcet.

## INTRODUCTION

Hereditary vitamin D Dependent rickets (HVDDR) is a rare autosomal recessive disorder. It is divided into type I and II, based on levels of calcitriol.^1^ They present as early-onset rickets with elevated alkaline phosphatase, hypocalcemia, hypophosphatemia, and hyperparathyroidism. We present a child with bone pain, stunted growth and teeth deformities. He has total alopecia suggesting lack of vitamin D receptor activity in keratinocytes which is associated with a severe form of disease.^2,3^ The elevated levels of 1,25-dihydroxy vitamin D points to possible resistance of the vitamin D receptor.^4^ Due to failure to respond, high doses of calcium and calcitriol, cinacalcet was added as an adjunct.^5^

## CASE REPORT

**Figure 1:**
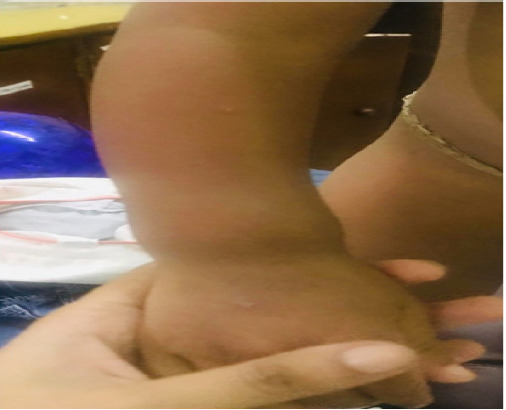
Wrist swelling before treatment.

A 2.5 years old male presented with a 2-year history of upper and lower extremities bone deformities and short stature. Hair loss was noticed at the age of 4 months and by the 7-month child was completely hairless (alopecia totalis). Prominent bilateral wrist joints were noted at around 8 months of age and by 1 year bowing of bilateral lower limbs were seen. Antenatal and birth events were unremarkable. There was no family history of short stature, skeletal disorders and consanguinity. He had delayed gross motor development. At the time of presentation at age 2, he had features of rickets namely bone pain, stunted growth and teeth deformities. There also was swelling in the wrist at the time of presentation (Figure 1).

**Figure 2:**
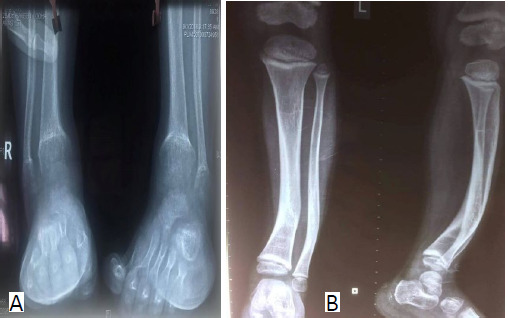
X-ray before treatment, A) Right leg and B) left leg at the time of presentation.

He had total alopecia. The anthropometric measurements were below the 3rd percentile and head circumference was at the 97th percentile. X-ray of right and left leg before treatment at the time of presentation there was the lack of provisional zone of calcification, widening of physis with metaphyseal cupping and fraying with generalized osteopenia (Figure 2).

The normal values as per the American Board of Internal Medicine Laboratory Test Reference Ranges were: Calcium: 8.8-10.8mg/dl, Phosphate: 3.8-6.6mg/ dl, ALP: 145-420IU/L, parathyroid hormone (PTH)(15-65ng/dl), 25(OH)D: >30ng/ml, 1,25(OH)2D: 20-80pg/ml. Biochemical parameters showed low levels of serum calcium, elevated levels of alkaline phosphatase, PTH and 1,25(OH)2D before initiating the treatment.

For initial treatment, a single dose of vitamin D 150000IU was given along with Tablet (Tab.) Calcitriol 3ug/day, Tab. Calcium 7gm/day. Tab cinacalcet 0.25mg/kg/day was added due to failure to respond (Table 1).

**Table 1:** Biochemical profile before and after Cinacalcet therapy.

Duration of treatment	Calcium (mg/dl)	Phospate (mg/dl)	^*^ALP (IU/L)	^||^PTH (ng/dl)	25(OH)D (ng/ml)	1,25(OH) 2D (pg/ml)	Treatment
Prior to admission	7.8	4.1	1002	602.7	37.5	480	Elemental calcium 7gm/day Calcitriol oral 3μg/day Vitamin D 3500IU/day
On first week of admission	7.8	4.1	808	500			Vitamin D 150000IU single dose Elemental calcium oral 7gm/day Calcitriol oral 3μg/day Cinacalcet 0.25mg/kg/day
After 1 months on cinacalcet	8	4.2	614	400			Elemental calcium oral 4gm/day Calcitriol oral 3μg/day Cinacalcet 0.4mg/kg/day
After 3 months on cinacalcet	8.6	4.3	432	227.6			Elemental calcium oral 3gm/day Calcitriol oral 2.25μg/day Cinacalcet 0.5mg/kg/day
After 6 months on cinacalcet	8.6	4.2	400	95			Elemental calcium oral 1.5gm/day Calcitriol oral 1μg/day Cinacalcet 0.4mg/kg/day
After 12 months on cinacalcet	9	5.2	234	43	52		Elemental calcium 1.5gm/day Calcitriol oral 0.5μg/day Cinacalcet 0.2mg/kg/day

* ALP: Alkaline phosphatase

|| PTH: Parathyroid hormone.

**Figure 3:**
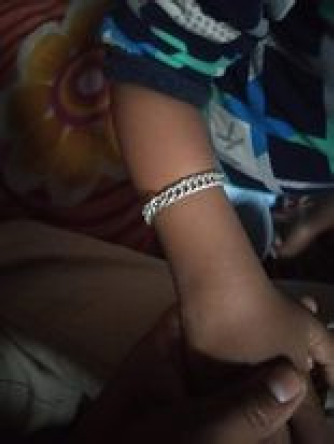
Resolution of wrist swelling after 1 year of treatment.

A significant decline in levels of Alkaline phosphatase and Parathyroid hormone was seen after the addition of cinacalcet. The dose was adjusted over 1 year and currently, he is receiving Tab. calcitriol D3 0.5μg/day, Tab Calcium 1.5gm/day and Tab cinacalcet 0.2mg/ kg/day. The child showed improvement with the decline of adenosine triphosphate (ATP) and PTH level in subsequent follow-up. Radiology showed improvement with the resolution of rachitic changes. In addition, the swelling of his hands subsided after treatment and he gained height (Figure 3).

## DISCUSSION

Vitamin D dependent Rickets type 2 (VDDR2) occurs due to mutations in the DNA-binding domain causing partial or total 1,25(OH)2D3 resistance and a severe phenotype of rickets.^6,7^ Akinci, et al. reported patients with mutations in the DNA-binding domain have alopecia, and those with mutations in the ligand-binding domain have no alopecia. The child we are reporting has total alopecia which supports the possibility of VDR mutation in the DNA-binding domain with severe hormone resistance.^8,9^

The early diagnosis of HVDDR is often misdiagnosed as alopecia, a dermatological condition as its early sign is alopecia. The child in our case had alopecia as early as 4 months, but the correct diagnosis was made only when rachitic changes became apparent. Nicolescu, et al. reported a similar scenario initially diagnosed as alopecia totalis and later correctly diagnosed when frontal bossing, relatively large head, widening of wrists, prominent genu varum was detected. In contrast, to the study published by Akinci, et al. and Nicolescu, et al. normo-phosphatemia was noted in our case.

As a consequence of failure to respond to the conventional doses of calcium, calcitriol and vitamin D initially a single dose of Vitamin D 150000IU was given to the patient and cinacalcet at a dose of 0.25mg/ kg/day was initiated along with oral calcium and calcitriol. With dose adjustment of cinacalcet over 1 year of treatment, improvements were observed in laboratory parameters with the decline of ALP and PTH level. The main metabolic characteristics of HVDDR are severe hypocalcaemia-induced secondary hyperparathyroidism and renal phosphate wasting which prolongs bone healing. The rationale behind the use of cinacalcet is its role in the reduction of PTH secretion by limiting phosphate wasting thus minimizing the duration required for bone healing.^3^ After cinacalcet initiation there was evidence of partial bone healing, significant improvement in radiographic findings, resolution of rachitic changes like the resolution of swelling of hands and bowing of legs and he gained height with a growth rate of 10cm/year was apparent. The conventional dose of calcium is not sufficient to suppress PTH despite a high oral dose of calcium in the case of HVDDR. Similarly, lab findings showed significant suppression of PTH level was seen only after doubling the dose of cinacalcet. Cinacalcet can also prevent complications like vitamin D toxicity, nephrocalcinosis and kidney damage due to the use of a high dose of calcium and calcitriol. When the dose of cinacalcet was titrated up from 0.25mg/kg/day up to 0.5mg/kg/day the dose of calcium significantly decreased from 7gm/day to 1.5gm/day showing a linear association between the doses.

Like other studies, our case showed a significant improvement after the addition of cinacalcet to conventional therapy of calcitriol and calcium. It also highlights the beneficial effect of using cinacalcet in achieving calcium-phosphate homeostasis through suppression of PTH secretion (secondary hyperparathyroidism) and in correcting the biochemical parameters and inducing radiological healing of rachitic deformities.^8,10^

## References

[1] Vitamin D-dependent rickets [Internet] (2017). https://medlineplus.gov/.

[2] Marx SJ, Spiegel AM, Brown EM, Gardner DG, Downs RW, Attie M (1978). A familial syndrome of decrease in sensitivity to 1,25-dihydroxyvitamin D. J Clin Endocrinol Metab.

[3] Goodyer PR, Kronick JB, Jequier S, Reade TM, Scriver CR (1987). Nephrocalcinosis and its relationship to treatment of hereditary rickets. J Pediatr.

[4] Brooks MH, Bell NH, Love L, Stern PH, Orfei E, Queener SF (1978). Vitamin-D-dependent rickets type II. Resistance of target organs to 1,25-dihydroxyvitamin D. N Engl J Med.

[5] Junaid Z, Patel J (2020). Cinacalcet [Internet].

[6] Malloy PJ, Wang J, Srivastava T, Feldman D (2010). Hereditary 1,25-dihydroxyvitamin D-resistant rickets with alopecia resulting from a novel missense mutation in the DNA-binding domain of the vitamin D receptor. Mol Genet Metab.

[7] Liberman UA, Samuel R, Halabe A, Kauli R, Edelstein S, Weisman Y (1980). End-organ resistance to 1,25-dihydroxycho-lecalciferol. Lancet.

[8] Akinci A, Dundar I, Kivilcim M (2017). The effectiveness of cinacalcet as an adjunctive therapy for hereditary 1,25 dihydroxyvitamin D3-resistant rickets. J Clin Res Pediatr Endocrinol.

[9] Rehman F, Dogra N, Wani MA (2019). Serum vitamin D levels and alopecia areata- a hospital based case-control study from North-India. Int J Trichology.

[10] Nicolescu RC, Lombet J, Cavalier E (2018). Vitamin D-resistant rickets and cinacalcet-one more favorable experience. Front Pediatr.

